# Genetic Variations of IL-12B, IL-12Rβ1, IL-12Rβ2 in Behcet's Disease and VKH Syndrome

**DOI:** 10.1371/journal.pone.0098373

**Published:** 2014-05-23

**Authors:** Xinyu Li, Lin Bai, Jing Fang, Shengping Hou, Qingyun Zhou, Hongsong Yu, Aize Kijlstra, Peizeng Yang

**Affiliations:** 1 The First Affiliated Hospital of Chongqing Medical University, Chongqing Key Laboratory of Ophthalmology and Chongqing Eye Institute, Chongqing, P. R. China; 2 University Eye Clinic Maastricht, Maastricht, The Netherlands; Kunming Institute of Zoology, Chinese Academy of Sciences, China

## Abstract

**Purpose:**

To investigate the associations of single nucleotide polymorphisms (SNPs) of three genes (IL-12B, IL-12Rβ1 and IL-12Rβ2) in Behcet's disease (BD) and Vogt-Koyanagi-Harada (VKH) syndrome in a Chinese Han population.

**Methods:**

A total of 806 BD cases, 820 VKH patients, and 1600 healthy controls were involved in this study. The first investigation included 400 BD patients, 400 VKH cases, and 600 healthy individuals. A second confirmatory study included a separate set of 406 BD patients, 420 VKH cases and another 1000 normal controls. Genotyping was carried out by PCR-restriction fragment length polymorphism assay and results were validated by using direct sequencing. The χ2 test was performed to compare the allele and genotype frequencies between cases and healthy controls.

**Results:**

This study comprised two phases. In the first phase study, a significantly increased frequency of the rs3212227/IL-12B genotype CC and C allele was found in BD patients as compared to controls (Bonferroni corrected p value (p_c_) = 0.009, OR 1.8; p_c_ = 0.024, OR 1.3, respectively). Moreover, the frequency of the C allele of rs3212227/IL-12B was also significantly increased in VKH patients (p_c_ = 0.012, OR 1.3, 95% CI 1.1 to 1.6). No associations were found for the other seven tested SNPs either in BD or VKH disease. The second study as well as the combined data confirmed the significant association of rs3212227/IL-12B with BD (CC genotype: combined p_c_ = 6.3×10^−7^, OR = 1.8; C allele: combined p_c_ = 2.0×10^−5^, OR = 1.3, respectively) and the C allele frequency of rs3212227/IL-12B as the risk factor to VKH patients (combined p_c_ = 2.5×10^−5^, OR 1.3, 95% CI 1.2 to 1.5).

**Conclusions:**

Our study revealed that the IL-12B gene is involved both in the susceptibility to BD as well as VKH syndrome.

## Introduction

Behcet's disease (BD) is considered to be a long-term, complex inflammatory disorder which is characterized by vasculitis involving multiple organs [1]. The clinical traits for BD comprise relapsing ulcerations in the oral cavity and genitals, erythema nodosum, ulcer of the colon and recurrent iritis [2], [3], [4]. Previous reports suggest that T cells play an important role in the pathogenesis of BD and an increased number of T cells is related with disease activity [5]. It has been shown that both Th1 and Th17 cells are crucial to the development of BD [6], [7].

Vogt–Koyanagi–Harada (VKH) syndrome is a multisystem disease which can often result in blindness. It is featured by a bilateral granulomatous panuveitis and concomitant extraocular symptoms, including vitiligo, alopecia, poliosis, impaired auditory sense and central nervous system lesions [8], [9]. An autoimmune reaction against melanocytes which may be triggered by infection or other factors, is thought to be involved in the pathogenesis of VKH disease [8]. We have shown that CD4 T cells and several inflammatory cytokines play an important role in the progress of the disorder [10]. Although the definite pathogenesis of the two diseases has not been determined, recent studies have shown an important role for an immunogenetic predisposition to both diseases. Various studies have demonstrated that several genes such as human leukocyte antigen (HLA)-B51, IL-23R, DHCR7, and TLR-2 are associated with BD [11], [12], [13], [14]. Furthermore, HLA-DR4, IL-17, STAT4 have been identified as predisposing factors for VKH syndrome [15], [16], [17].

Interleukins are types of cytokine signaling molecules in the immune system and play important roles in regulating the activities of lymphocytes that are responsible for immunity. Interleukin-12B (IL-12B) encodes the key subunit IL-12p40 which is the common component of IL-12 and IL-23. The bioactive subunit is generated mainly by activated macrophages and dendritic cells in response to pathogenic or inflammatory factors [18], [19]. IL-12p40 deficiency leads to decreased migration of dendritic cells and diminished ability to activate naive T cells [20]. It also has considerable roles in the function of the Th1 and Th17 cells [21], [22], the two significant kinds of T-helper subsets involved in many important immunological processes. Morahan et al [23] have revealed that differences in the degree of IL-12p40 production may have significant impact on T cell reactivity which plays an essential role in immune-related diseases. Recent studies indicated that a genetic aberration of the IL-12B gene is associated with several autoimmune diseases such as ankylosing spondylitis (AS) and psoriasis [24], [25]. Interleukin-12-receptor-β1 (IL-12Rβ1) is a receptor chain which is shared by IL-12 and IL-23. IL-12Rβ1 is critical for the function and development of memory CD4 T cells [26]. The IL-12Rβ1-dependent pathway is essential for the differentiation and proliferation of human Th17 cell subsets [27]. IL-12Rβ1 knockout mice have been shown to be resistant to Experimental Autoimmune Encephalomyelitis (EAE) [28]. Mutations in the IL-12Rβ1 gene have been shown to be associated with immune disorders [29]. IL-12Rβ2 is the other subunit of the IL-12 receptors. IL-12Rβ2 is essential in Th1 development and is also associated with IL-17 expression [30], [31]. Furthermore, IL-12Rβ2 deficiency can lead to a diminished recruitment of activated T cells and an abnormal expression of inflammatory cytokines [32].

Given the fact that these related genes (IL-12B, IL-12Rβ1 and IL-12Rβ2) play an important role in Th1 and Th17 related immune responses, we wanted to verify the potential evidence of an association of polymorphisms in these genes with uveitis. We chose BD and VKH as representative examples of uveitis in view of the fact that a sufficiently large sample size could be obtained. Eight SNPs of the three genes (IL-12B, IL-12Rβ1 and IL-12Rβ2) were selected for our study, whereby the choice was dictated by previous studies showing a definite association with other autoimmune diseases.

## Materials and Methods

### Study Population

This case control study was composed of two phases. In the first phase we tested 400 BD patients, 400 VKH syndrome patients and 600 healthy controls. In the second stage, which was separate from the first one, another set of 406 BD patients, 420 VKH cases and another 1000 healthy subjects were included. All the individuals had visited the uveitis clinic at the First Affiliated Hospital of Chongqing Medical University, Chongqing, China and Zhongshan Ophthalmic Center, Sun Yat-sen University Guangzhou, China, between April 2005 and December 2013. The healthy controls were geographically and ethnically matched with the patients. All the subjects involved in this study were Han Chinese. The diagnostic criteria for BD and VKH syndrome were precisely followed as described by the International Study Group for BD [33] and First International Workshop for VKH [34]. The clinical features of the BD and VKH disease patients included in our study are presented in table 1.

**Table 1:** Clinical features of the investigated BD and VKH syndrome patients.

Extraocular findings	Total	%
**Patients with BD**	806	
Mean age ± SD	33.7±8.9	
Male	699	86.7
Female	107	13.3
Uveitis	806	100
Oral ulcer	806	100
Genital ulcer	460	57.1
Skin lesions	610	75.7
Arthritis	119	14.8
Positive pathergy test	153	19.0
**Patients with VKH**	820	
Mean age ± SD	39.0±13.8	
Male	438	53.4
Female	382	46.6
Uveitis	820	100
Headache	325	39.6
Tinnitus	249	30.4
Vitiligo	152	18.5
Poliosis	303	37.0
**Normal controls**	820	
Mean age ± SD	39.7±10.7	
Male	869	54.3
Female	731	45.7

BD, Behcet's disease; VKH, Vogt–Koyanagi–Harada syndrome.

The ethics committee of the First Affiliated Hospital of Chongqing Medical University approved the study protocol (Permit Number: 2009-201008) and all procedures followed the tenets of the Declaration of Helsinki. All the participants provided their written informed consent to participate in this study. We recorded all the participant consents in the book which kept the whole consents in it. The study was registered in the Chinese Clinical Trial Registry. The registration number is ChiCTR-CCC-12002184.

### SNP selection and genotyping

**Figure 1:**
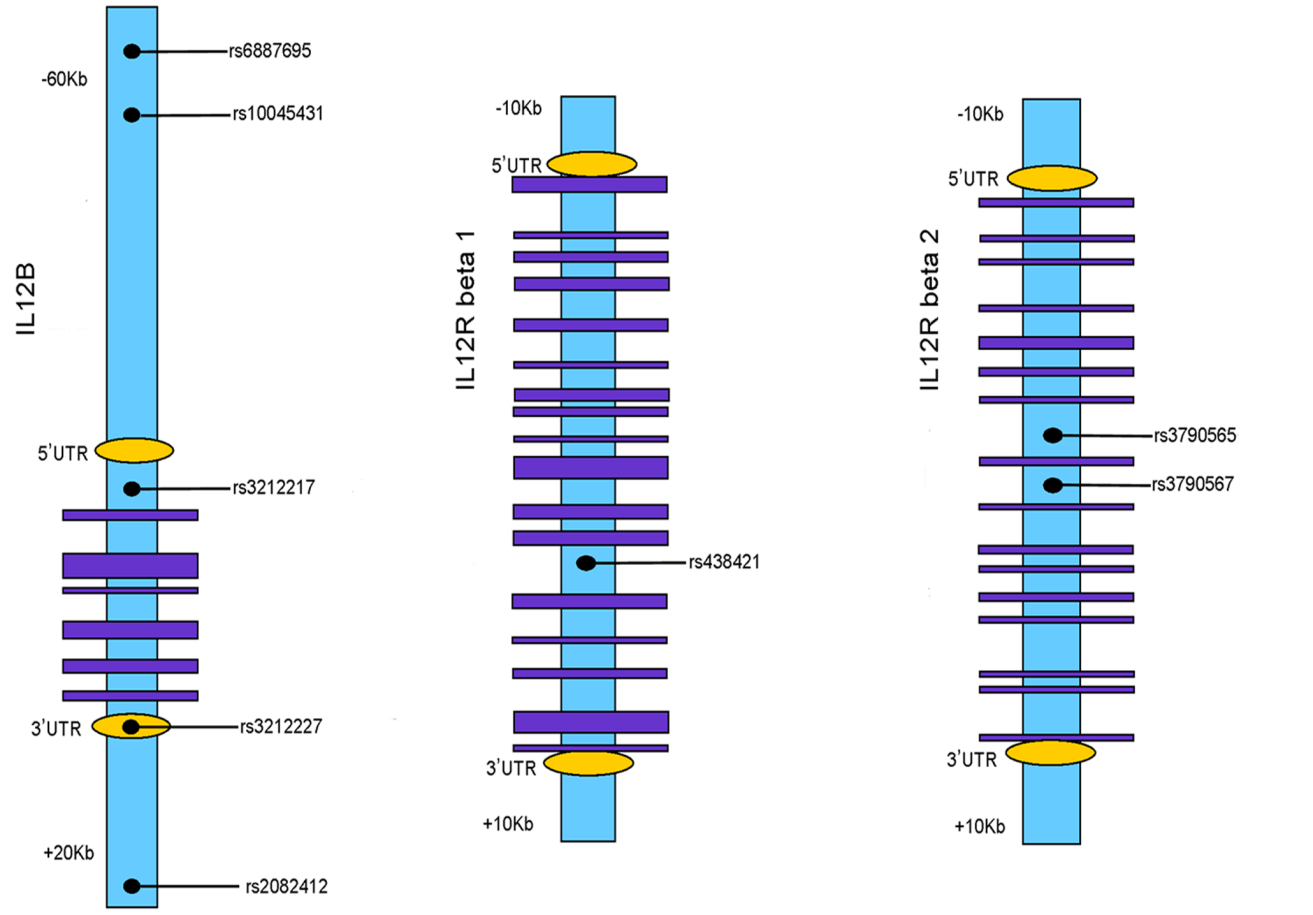
The sketch of gene structure of the selected SNPs.

According to previous reported associations of the three IL-12 related genes (IL-12B, IL-12Rβ1 and IL-12Rβ2) polymorphisms with autoimmune disorders, five SNPs of IL-12B (rs3212227, rs6887695, rs3212217, rs10045431, rs2082412), one SNP of IL-12Rβ1 (rs438421) and two SNPs for IL-12Rβ2 (rs3790565, rs3790567) were chosen for our study to detect the potential relevance of these genes with our two uveitis entities. The selected SNPs and a sketch of the gene structure are shown in Figure 1. Genomic DNA of the subjects was extracted with the QIA amp DNA Blood Mini Kit (Qiagen, Valencia, CA). After this, the extracted DNA samples were stored at -20°C.

The appropriate primers used to amplify the corresponding target DNA sequence by PCR and corresponding restriction enzymes are shown in Table 2. Five percent agarose gels were used to separate the digestion products and stained with GoldView TM (SBS Genetech, Beijing, China). To ensure the validity of the method for our study, 20% of all samples were stochastically selected for direct sequencing by Sangon Biotech Company (Shanghai, China).

**Table 2:** Primers and restriction enzymes used for restricted fragment length polymorphisms analysis of the IL12B, IL-12Rβ1 and IL-12Rβ2 genes.

Gene	SNP number	Primers	Restriction enzyme
IL-12B	rs3212227	5′GACACAACGGAATAGACCCAAAAAG3′	TaqI
		5′GGCAACTTGAGAGCTGGAAAATCT 3′	
	rs6887695	5′GAGAGAAGCAGTGTAGTGTAGTGAT3′	BclI
		5′GAACAGAGAGGGGAAGCAAC3′	
	rs10045431	5′CAGGGTAGTGGGTATTGTCCTAAGTAT3′	HinfI
		5′GGGTAGTGGGTATTGTCCTAAGTATGC3	
	rs3212217	5′ TAAAGTACATAGCAAGGTCCGCCTCG3′	XhoI
		5′ AGCACCCATGGTCTGGAGGAAA3′	
	rs2082412	5′ TCTCCTCTCCAGAGTGTTCCAGTAA3′	SduI
		5′CGAGTGAAATTGTACAAATGCAAAGTG3′	
IL-12Rβ1	rs438421	5′CACCACCGACATGCACTTGAA3′	StyI
		5′ AGGGATGACTGTCTCCATTCCAC3′	
IL-12Rβ2	rs3790565	5′ GGTCTATTGTAAATCCCTAGTGAGG 3′	TaqI
		5′GGGAGATAGAGTTGAATATTGCATC3′	
	rs3790567	5′GAAAAGCCCTCCTCTCTACCTTAA3′	AflII
		5′CTTGTGGTCTTCACATTTGTCTTG3′	

### Statistical analysis

The χ2 test was applied for the evaluation of the Hardy-Weinberg equilibrium. Direct counting was used to assess the genotype frequencies. The χ2 test using SPSS (version 13.0) was used for the comparison of genotype and allele frequencies between patients and healthy controls. Bonferroni correction was carried out for the correction of multiple comparisons by multiplying p values with the number of analyses performed. A statistical significant difference was considered when the p_c_<0.05.

## Results

### Clinical features of BD/VKH

The clinical characteristics of the enrolled BD patients and VKH cases are shown in Table 1. BD and VKH disease usually appear in the young and middle aged population. The features for BD comprise relapsing ulcerations in the oral cavity and genital region, skin lesions and arthritis. Extraocular symptoms of VKH syndrome include vitiligo, alopecia, poliosis, impaired auditory sense and central nervous system lesions. Details of the enrolled controls are also shown in Table 1.

### Associations of the IL-12B gene polymorphisms with susceptibility to BD

In the first part of this case-control study, eight SNPs of the three IL-12 related genes (IL-12B, IL-12Rβ1 and IL-12Rβ2) were genotyped successfully in 400 BD patients and 600 healthy controls. The obtained data of healthy subjects were in agreement with the Hardy-Weinberg equilibrium. Frequencies of the rs3212227/IL-12B genotype CC and C allele were significantly higher in the BD patients (p_c_ = 0.009, OR 1.8, 95% CI 1.3 to 2.4; p_c_ = 0.024, OR 1.3, 95% CI 1.1 to 1.6, respectively) compared with the normal controls. To confirm the result, a second set of patients was used to replicate the associated SNP rs3212227/IL-12B using another 406 BD samples and another set of 1000 healthy controls. In the second stage, the frequencies of the rs3212227/IL-12B genotype CC and C allele were also significantly higher in BD cases (p_c_ = 3.5×10^−4^, OR = 1.8, 95% CI 1.4 to 2.4; p_c_ = 0.002, OR = 1.4, 95% CI 1.2 to 1.6, respectively) than that observed in the control group. Subsequently, we analyzed the combined data of the two studies, which showed a consistent association of the CC genotype and C allele of rs3212227/IL-12B with BD (p_c__comb_ = 6.3×10^−7^, OR = 1.8, 95% CI 1.5 to 2.2; p_c__comb_ = 2.0×10^−5^, OR = 1.3, 95% CI 1.2 to 1.5, respectively). There was no significant association between BD and the other tested SNPs (four of IL-12B, one of IL-12Rβ1 and two of IL-12Rβ2 (Table 3). Since several organs may be affected by BD, a further study was undertaken to explore the association of these SNPs with the clinical manifestations in the BD cases. No associations between tested gene polymorphisms with the separate features of BD could be detected (data not shown).

**Table 3:** Frequencies of genotypes and alleles of IL12B, IL-12Rβ1 and IL-12Rβ2 polymorphisms in patients with BD and controls.

SNPs	Stage	Genotype	Case	Control	P value	P_c_ value	OR(95%CI)
		Allele	n(freq)	n(freq)			
	First	A A	114(0.285)	199(0.332)	0.119	NS	0.803(0.610–1.058)
		A C	188(0.470)	308(0.513)	0.179	NS	0.841(0.653–1.083)
		C C	98(0.245)	93(0.155)	3.90×10^−4^	0.009	1.769(1.288–2.430)
		A	416(0.520)	706(0.588)	0.003	0.024	0.758(0.633–0.908)
		C	384(0.480)	494(0.412)	0.003	0.024	1.319(1.102–1.580)
rs3212227	Replication	A A	110(0.271)	323(0.323)	0.055	NS	0.779(0.603–1.006)
(IL-12B)		A C	190(0.468)	516(0.516)	0.103	NS	0.825(0.655–1.040)
		C C	106(0.261)	161(0.161)	1.45×10^−5^	3.48×10^−4^	1.841(1.394–2.432)
		A	410(0.505)	1162(0.581)	2.31×10^−4^	0.002	0.736(0.624–0.866)
		C	402(0.495)	838(0.419)	2.31×10^−4^	0.002	1.360(1.154–1.601)
	Combined	A A	224(0.278)	522(0.326)	0.016	NS	0.795(0.660–0.958)
		A C	378(0.469)	824(0.515)	0.033	NS	0.832(0.702–0.985)
		C C	204(0.253)	254(0.159)	2.63×10^−8^	6.31×10^−7^	1.796(1.459–2.211)
		A	826(0.512)	1868(0.584)	2.53×10^−6^	2.02×10^−5^	0.749(0.664–0.845)
		C	786(0.488)	1332(0.416)	2.53×10^−6^	2.02×10^−5^	1.334(1.183–1.505)
rs6887695	First	C C	62(0.155)	87(0.145)	0.664	NS	1.082(0.760–1.540)
(IL-12B)		C G	199(0.497)	303(0.505)	0.540	NS	0.924(0.719–1.188)
		G G	139(0.348)	210(0.350)	0.935	NS	0.989(0.758–1.290)
		C	323(0.404)	477(0.398)	0.780	NS	1.026(0.855–1.232)
		G	477(0.596)	723(0.602)	0.780	NS	0.974(0.812–1.169)
rs10045431	First	A C	54(0.135)	88(0.147)	0.605	NS	0.908(0.630–1.308)
(IL-12B)		C C	346(0.865)	512(0.853)	0.605	NS	1.101(0.764–1.587)
		A	54(0.068)	88(0.073)	0.619	NS	0.915(0.644–1.300)
		C	746(0.932)	1112(0.927)	0.619	NS	1.093(0.769–1.553)
rs3212217	First	C C	83(0.207)	101(0.168)	0.117	NS	1.294(0.937–1.786)
(IL-12B)		C G	214(0.535)	317(0.528)	0.836	NS	1.027(0.797–1.324)
		G G	103(0.258)	182(0.304)	0.095	NS	0.785(0.591–1.043)
		C	380(0.475)	519(0.432)	0.061	NS	1.187(0.992–1.421)
		G	420(0.525)	681(0.568)	0.061	NS	0.842(0.704–1.008)
rs2082412	First	A A	72(0.180)	104(0.173)	0.786	NS	1.047(0.752–1.458)
(IL-12B)		A G	210(0.525)	311(0.518)	0.836	NS	1.027(0.797–1.323)
		G G	118(0.295)	185(0.309)	0.653	NS	0.939(0.712–1.237)
		A	354(0.443)	519(0.432)	0.659	NS	1.041(0.870–1.247)
		G	446(0.557)	681(0.568)	0.659	NS	0.960(0.802–1.150)
rs438421	First	A A	35(0.087)	66(0.110)	0.247	NS	0.776(0.504–1.194)
(IL-12 Rβ1)		A G	178(0.445)	281(0.468)	0.468	NS	0.910(0.706–1.174)
		G G	187(0.468)	253(0.422)	0.153	NS	1.204(0.933–1.553)
		A	248(0.310)	413(0.344)	0.112	NS	0.856(0.707–1.037)
		G	552(0.690)	787(0.656)	0.112	NS	1.168(0.965–1.414)
rs3790565	First	C C	17(0.043)	19(0.032)	0.368	NS	1.357(0.697–2.644)
(IL-12 Rβ2)		C T	146(0.365)	194(0.323)	0.173	NS	1.203(0.922–1.569)
		T T	237(0.592)	387(0.645)	0.093	NS	0.800(0.617–1.038)
		C	180(0.225)	232(0.193)	0.086	NS	1.211(0.973–1.508)
		T	620(0.775)	968(0.807)	0.086	NS	0.826(0.663–1.028)
rs3790567	First	A A	26(0.065)	25(0.042)	0.100	NS	1.599(0.909–2.811)
(IL-12 Rβ2)		A G	162(0.405)	232(0.387)	0.561	NS	1.080(0.834–1.398)
		G G	212(0.530)	343(0.571)	0.194	NS	0.845(0.655–1.090)
		A	214(0.268)	282(0.235)	0.099	NS	1.189(0.968–1.460)
		G	586(0.733)	918(0.765)	0.099	NS	0.841(0.685–1.033)

SNP, single-nucleotide polymorphism; OR, odds ratio; CI, confidence interval; NS, no significant different; freq, frequency; p_c_, p Bonferroni correction.

### IL-12B gene confers susceptibility to VKH syndrome

In the first study we included 400 VKH patients and 600 normal controls. The samples of patients and controls were genotyped for the eight SNPs mentioned above. We found that the C allele frequency of rs3212227/IL-12B was significantly increased in VKH patients (p_c_ = 0.012, OR 1.3, 95% CI 1.1 to 1.6). Based on this result, we performed a confirmatory study with another set of 420 VKH cases and 1000 healthy individuals. Consistent with the result in the first study, the C allele frequency of rs3212227/IL-12B was markedly increased in VKH patients as compared to normal subjects (p_c_ = 0.046, OR 1.3, 95% CI 1.1 to 1.6). Subsequently, we analyzed the combined data of all the cases and controls. The data confirmed the C allele frequency of rs3212227/IL-12B as the risk factor for VKH (p_c_ = 2.5×10^−5^, OR 1.3, 95% CI 1.2 to 1.5). No statistically significant association was found between VKH and the other seven SNPs tested (Table 4). In addition, no associations could be detected between the separate VKH disease characteristics and the investigated SNPs (data not shown).

**Table 4:** Frequencies of genotypes and alleles of IL12B, IL-12Rβ1 and IL-12Rβ2 polymorphisms in patients with VKH patients and controls.

SNPs	Stage	Genotype	Case	Control	P value	P_c_ value	OR(95%CI)
		Allele	n(freq)	n(freq)			
	First	A A	94(0.235)	199(0.332)	0.001	0.024	0.619(0.465–0.825)
		A C	225(0.562)	308(0.513)	0.127	NS	1.219(0.945–1.572)
		C C	81(0.203)	93(0.155)	0.052	NS	1.384(0.996–1.924)
		A	413(0.516)	706(0.588)	0.0015	0.012	0.747(0.624–0.894)
		C	387(0.484)	494(0.412)	0.0015	0.012	1.339(1.118–1.603)
rs3212227	Replication	A A	98(0.233)	323(0.323)	7.34×10^−4^	0.018	0.638(0.491–0.829)
(IL-12B)		A C	233(0.555)	516(0.516)	0.182	NS	1.169(0.930–1.469)
		C C	89(0.212)	161(0.161)	0.022	NS	1.401(1.050–1.870)
		A	429(0.511)	1162(0.581)	5.73×10^−4^	0.046	0.753(0.640–0.885)
		C	411(0.489)	838(0.419)	5.73×10^−4^	0.046	1.328(1.130–1.562)
	Combined	A A	192(0.234)	522(0.326)	2.57×10^−6^	6.17×10^−5^	0.631(0.521–0.765)
		A C	458(0.559)	824(0.515)	0.042	NS	1.191(1.006–1.411)
		C C	170(0.207)	254(0.159)	0.003	NS	1.386(1.117–1.719)
		A	842(0.513)	1868(0.584)	3.08×10^−6^	2.46×10^−5^	0.752(0.668–0.848)
		C	798(0.487)	1332(0.416)	3.08×10^−6^	2.46×10^−5^	1.329(1.179–1.498)
rs6887695	First	C C	61(0.152)	87(0.145)	0.744	NS	1.061(0.744–1.513)
(IL-12B)		C G	216(0.541)	303(0.505)	0.278	NS	1.151(0.893–1.483)
		G G	123(0.307)	210(0.350)	0.162	NS	0.825(0.629–1.081)
		C	338(0.422)	477(0.398)	0.265	NS	1.109(0.925–1.330)
		G	462(0.578)	723(0.602)	0.265	NS	0.902(0.752–1.082)
rs10045431	First	A C	56(0.140)	88(0.147)	0.769	NS	0.947(0.660–1.360)
(IL-12B)		C C	344(0.860)	512(0.853)	0.769	NS	1.056(0.735–1.516)
		A	56(0.070)	88(0.073)	0.778	NS	0.951(0.672–1.347)
		C	744(0.930)	1112(0.927)	0.778	NS	1.051(0.743–1.489)
rs3212217	First	C C	77(0.193)	101(0.168)	0.328	NS	1.178(0.849–1.635)
(IL-12B)		C G	213(0.532)	317(0.528)	0.897	NS	1.017(0.789–1.310)
		G G	110(0.275)	182(0.304)	0.334	NS	0.871(0.658–1.153)
		C	367(0.459)	519(0.432)	0.247	NS	1.112(0.929–1.331)
		G	433(0.541)	681(0.568)	0.247	NS	0.899(0.751–1.076)
rs2082412	First	A A	90(0.225)	104(0.173)	0.100	NS	1.306(0.950–1.797)
(IL-12B)		A G	196(0.490)	311(0.518)	0.570	NS	0.929(0.721–1.197)
		G G	114(0.285)	185(0.309)	0.430	NS	0.894(0.677–1.180)
		A	376(0.470)	519(0.432)	0.152	NS	1.140(0.953–1.365)
		G	424(0.530)	681(0.568)	0.152	NS	0.877(0.733–1.050)
rs438421	First	A A	38(0.095)	66(0.110)	0.447	NS	0.849(0.558–1.294)
(IL-12 Rβ1)		A G	189(0.472)	281(0.468)	0.897	NS	1.017(0.789–1.310)
		G G	173(0.433)	253(0.422)	0.734	NS	1.045(0.809–1.350)
		A	265(0.331)	413(0.344)	0.550	NS	0.944(0.781–1.141)
		G	535(0.669)	787(0.656)	0.550	NS	1.059(0.877–1.280)
rs3790565	First	C C	12(0.030)	19(0.032)	0.882	NS	0.946(0.454–1.970)
(IL-12Rβ2)		C T	128(0.320)	194(0.323)	0.912	NS	0.985(0.751–1.291)
		T T	260(0.650)	387(0.645)	0.871	NS	1.022(0.784–1.332)
		C	152(0.190)	232(0.193)	0.853	NS	0.979(0.780–1.229)
		T	648(0.810)	968(0.807)	0.853	NS	1.022(0.814–1.283)
rs3790567	First	A A	24(0.060)	25(0.042)	0.188	NS	1.468(0.826–2.609)
(IL-12Rβ2)		A G	157(0.393)	232(0.387)	0.853	NS	1.025(0.791–1.328)
		G G	219(0.547)	343(0.571)	0.450	NS	0.907(0.703–1.170)
		A	205(0.256)	282(0.235)	0.278	NS	1.122(0.912–1.380)
		G	595(0.744)	918(0.765)	0.278	NS	0.892(0.725–1.097)

SNP, single-nucleotide polymorphism; OR, odds ratio; CI, confidence interval; NS, no significant different; freq, frequency; p_c_, p Bonferroni correction.

**Figure 2:**
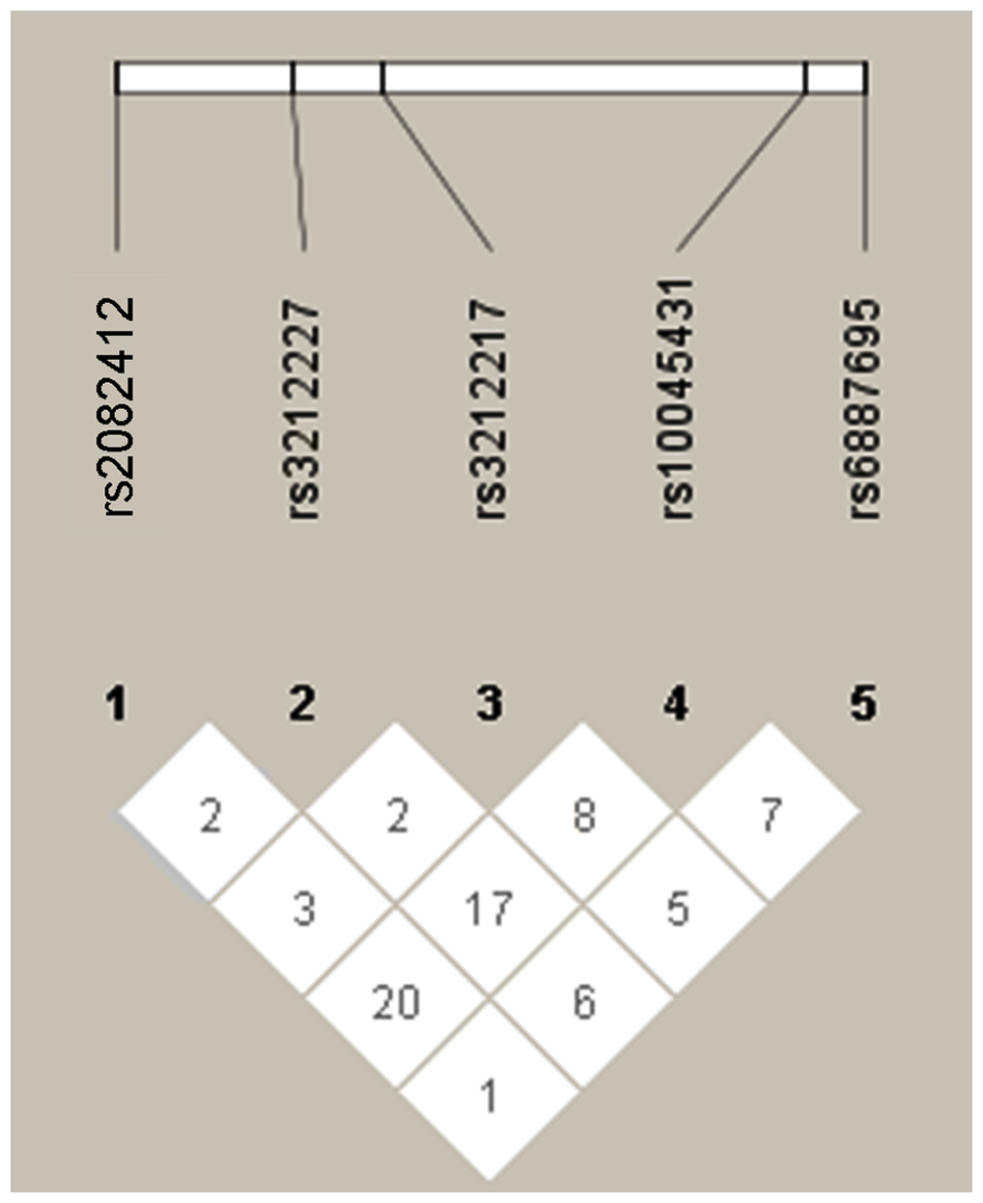
Pair-wise linkage disequilibrium values of IL-12B SNPs in a Chinese Han population. Linkage Disequilibrium (LD) block was estimated for IL-12B gene locus using our data. The pair-wise D' Values are shown in blocks.

We analyzed the data of five SNPs of IL-12B for LD (Linkage Disequilibrium), and found that there was no linkage between these loci (Figure 2). In addition, we performed a comparison between BD and VKH, and between patients (BD and VKH) and controls. The data were in agreement with the present results both in BD and VKH group (Table 5 and Table 6, respectively).

**Table 5:** Comparison of frequencies of genotypes and alleles of IL12B, IL-12Rβ1 and IL-12Rβ2 polymorphisms between BD and VKH cases.

SNPs	Stage	Genotype	BD Case	VKH Case	P value	P_c_ value	BD vs VKH
		Allele	n(freq)	n(freq)			OR(95%CI)
	First	A A	114(0.285)	94(0.235)	0.107	NS	1.298(0.945–1.782)
		A C	188(0.470)	225(0.562)	0.009	NS	0.690(0.522–0.911)
		C C	98(0.245)	81(0.203)	0.149	NS	1.278(0.915–1.784)
		A	416(0.520)	413(0.516)	0.881	NS	1.015(0.834–1.235)
		C	384(0.480)	387(0.484)	0.881	NS	0.985(0.810–1.199)
rs3212227	Replication	A A	110(0.271)	98(0.233)	0.213	NS	1.221(0.891–1.673)
(IL-12B)		A C	190(0.468)	233(0.555)	0.013	NS	0.706(0.537–0.928)
		C C	106(0.261)	89(0.212)	0.096	NS	1.314(0.952–1.814)
		A	410(0.505)	429(0.511)	0.814	NS	0.977(0.806–1.185)
		C	402(0.495)	411(0.489)	0.814	NS	1.023(0.844–1.241)
	Combined	A A	224(0.278)	192(0.234)	0.043	NS	1.259(1.007–1.574)
		A C	378(0.469)	458(0.559)	3.03×10^−4^	0.007	0.698(0.574–0.849)
		C C	204(0.253)	170(0.207)	0.028	NS	1.296(1.028–1.634)
		A	826(0.512)	842(0.513)	0.954	NS	0.996(0.868–1.143)
		C	786(0.488)	798(0.487)	0.954	NS	1.004(0.875–1.152)
rs6887695	First	C C	62(0.155)	61(0.152)	0.922	NS	1.019(0.694–1.497)
(IL-12B)		C G	199(0.497)	216(0.541)	0.229	NS	0.843(0.639–1.113)
		G G	139(0.348)	123(0.307)	0.228	NS	1.199(0.892–1.612)
		C	323(0.404)	338(0.422)	0.446	NS	0.926(0.759–1.129)
		G	477(0.596)	462(0.578)	0.446	NS	1.080(0.885–1.318)
rs10045431	First	A C	54(0.135)	56(0.140)	0.837	NS	0.959(0.641–1.434)
(IL-12B)		C C	346(0.865)	344(0.860)	0.837	NS	1.043(0.697–1.560)
		A	54(0.068)	56(0.070)	0.843	NS	0.962(0.653–1.417)
		C	746(0.932)	744(0.930)	0.843	NS	1.040(0.706–1.532)
rs3212217	First	C C	83(0.207)	77(0.193)	0.596	NS	1.098(0.777–1.553)
(IL-12B)		C G	214(0.535)	213(0.532)	0.943	NS	1.010(0.765–1.334)
		G G	103(0.258)	110(0.275)	0.576	NS	0.914(0.668–1.251)
		C	380(0.475)	367(0.459)	0.515	NS	1.067(0.877–1.299)
		G	420(0.525)	433(0.541)	0.515	NS	0.937(0.770–1.140)
rs2082412	First	A A	72(0.180)	90(0.225)	0.113	NS	0.756(0.535–1.069)
(IL-12B)		A G	210(0.525)	196(0.490)	0.322	NS	1.150(0.872–1.518)
		G G	118(0.295)	114(0.285)	0.755	NS	1.050(0.773–1.425)
		A	354(0.443)	376(0.470)	0.269	NS	0.895(0.735–1.090)
		G	446(0.557)	424(0.530)	0.269	NS	1.117(0.918–1.360)
rs438421	First	A A	35(0.087)	38(0.095)	0.713	NS	0.913(0.564–1.479)
(IL-12 Rβ1)		A G	178(0.445)	189(0.472)	0.435	NS	0.895(0.678–1.182)
		G G	187(0.468)	173(0.433)	0.320	NS	1.152(0.872–1.522)
		A	248(0.310)	265(0.331)	0.362	NS	0.907(0.735–1.119)
		G	552(0.690)	535(0.669)	0.362	NS	1.103(0.894–1.360)
rs3790565	First	C C	17(0.043)	12(0.030)	0.344	NS	1.435(0.676–3.045)
(IL-12 Rβ2)		C T	146(0.365)	128(0.320)	0.180	NS	1.221(0.912–1.637)
		T T	237(0.592)	260(0.650)	0.094	NS	0.783(0.588–1.042)
		C	180(0.225)	152(0.190)	0.084	NS	1.238(0.971–1.577)
		T	620(0.775)	648(0.810)	0.084	NS	0.808(0.634–1.030)
rs3790567	First	A A	26(0.065)	24(0.060)	0.770	NS	1.089(0.614–1.932)
(IL-12 Rβ2)		A G	162(0.405)	157(0.393)	0.718	NS	1.054(0.794–1.398)
		G G	212(0.530)	219(0.547)	0.620	NS	0.932(0.706–1.231)
		A	214(0.268)	205(0.256)	0.609	NS	1.060(0.848–1.325)
		G	586(0.733)	595(0.744)	0.609	NS	0.943(0.755–1.179)

SNP, single-nucleotide polymorphism; OR, odds ratio; CI, confidence interval; NS, no significant different; freq, frequency; p_c_, p Bonferroni correction.

**Table 6:** Comparison of frequencies of genotypes and alleles of IL12B, IL-12Rβ1 and IL-12Rβ2 polymorphisms between cases (BD + VKH) and controls.

SNPs	Stage	Genotype	BD+VKH	Control	P value	P_c_ value	OR(95%CI)
		Allele	n(freq)	n(freq)			
	First	A A	208(0.260)	199(0.332)	0.003	NS	0.708(0.561–0.893)
		A C	413(0.516)	308(0.513)	0.914	NS	1.012(0.819–1.250)
		C C	179(0.224)	93(0.155)	0.001	0.024	1.571(1.192–2.072)
		A	829(0.518)	706(0.588)	2.21×10^−4^	0.002	0.752(0.647–0.875)
		C	771(0.482)	494(0.412)	2.21×10^−4^	0.002	1.329(1.143–1.546)
rs3212227	Replication	A A	208(0.252)	323(0.323)	8.57×10^−4^	0.021	0.705(0.574–0.866)
(IL-12B)		A C	423(0.512)	516(0.516)	0.868	NS	0.985(0.819–1.184)
		C C	195(0.236)	161(0.161)	5.56×10^−5^	0.001	1.610(1.276–2.033)
		A	839(0.508)	1162(0.581)	9.89×10^−6^	7.91×10^−5^	0.744(0.653–0.849)
		C	813(0.492)	838(0.419)	9.89×10^−6^	7.91×10^−5^	1.344(1.179–1.532)
	Combined	A A	416(0.256)	522(0.326)	1.07×10^−5^	2.57×10^−4^	0.710(0.609–0.827)
		A C	836(0.514)	824(0.515)	0.961	NS	0.997(0.868–1.144)
		C C	374(0.230)	254(0.159)	3.20×10^−7^	7.68×10^−6^	1.583(1.326–1.889)
		A	1668(0.513)	1868(0.584)	1.09×10^−8^	8.72×10^−8^	0.751(0.681–0.828)
		C	1584(0.487)	1332(0.416)	1.09×10^−8^	8.72×10^−8^	1.332(1.207–1.469)
rs6887695	First	C C	123(0.154)	87(0.145)	0.650	NS	1.071(0.796–1.443)
(IL-12B)		C G	415(0.519)	303(0.505)	0.610	NS	1.057(0.855–1.306)
		G G	262(0.327)	210(0.350)	0.378	NS	0.904(0.723–1.131)
		C	661(0.413)	477(0.398)	0.405	NS	1.067(0.916–1.243)
		G	939(0.587)	723(0.602)	0.405	NS	0.937(0.805–1.092)
rs10045431	First	A C	110(0.138)	88(0.147)	0.626	NS	0.928(0.685–1.255)
(IL-12B)		C C	690(0.863)	512(0.853)	0.626	NS	1.078(0.797–1.459)
		A	110(0.069)	88(0.073)	0.640	NS	0.933(0.697–1.248)
		C	1490(0.931)	1112(0.927)	0.640	NS	1.072(0.801–1.434)
rs3212217	First	C C	160(0.200)	101(0.168)	0.132	NS	1.235(0.938–1.626)
(IL-12B)		C G	427(0.534)	317(0.528)	0.841	NS	1.022(0.827–1.263)
		G G	213(0.266)	182(0.304)	0.127	NS	0.833(0.659–1.053)
		C	747(0.467)	519(0.432)	0.071	NS	1.149(0.988–1.336)
		G	853(0.533)	681(0.568)	0.071	NS	0.870(0.749–1.012)
rs2082412	First	A A	162(0.202)	104(0.173)	0.169	NS	1.211(0.922–1.591)
(IL-12B)		A G	406(0.508)	311(0.518)	0.688	NS	0.958(0.775–1.183)
		G G	232(0.290)	185(0.309)	0.458	NS	0.916(0.727–1.154)
		A	730(0.456)	519(0.432)	0.211	NS	1.101(0.947–1.280)
		G	870(0.544)	681(0.568)	0.211	NS	0.908(0.781–1.056)
rs438421	First	A A	73(0.091)	66(0.110)	0.246	NS	0.812(0.572–1.154)
(IL-12 Rβ1)		A G	367(0.459)	281(0.468)	0.722	NS	0.962(0.778–1.190)
		G G	360(0.450)	253(0.422)	0.290	NS	1.122(0.906–1.389)
		A	513(0.321)	413(0.344)	0.190	NS	0.899(0.767–1.054)
		G	1087(0.679)	787(0.656)	0.190	NS	1.112(0.949–1.303)
rs3790565	First	C C	29(0.036)	19(0.032)	0.641	NS	1.150(0.639–2.072)
(IL-12 Rβ2)		C T	274(0.343)	194(0.323)	0.452	NS	1.090(0.871–1.365)
		T T	497(0.621)	387(0.645)	0.362	NS	0.903(0.725–1.125)
		C	332(0.208)	232(0.193)	0.355	NS	1.092(0.906–1.318)
		T	1268(0.792)	968(0.807)	0.355	NS	0.915(0.759–1.104)
rs3790567	First	A A	50(0.063)	25(0.042)	0.087	NS	1.533(0.937–2.508)
(IL-12 Rβ2)		A G	319(0.399)	232(0.387)	0.647	NS	1.052(0.847–1.307)
		G G	431(0.538)	343(0.571)	0.220	NS	0.875(0.707–1.083)
		A	419(0.262)	282(0.235)	0.104	NS	1.155(0.971–1.374)
		G	1181(0.738)	918(0.765)	0.104	NS	0.866(0.728–1.030)

SNP, single-nucleotide polymorphism; OR, odds ratio; CI, confidence interval; NS, no significant different; freq, frequency; p_c_, p Bonferroni correction.

## Discussion

This study shows that IL-12B gene polymorphisms are associated with two uveitis entities known as BD and VKH syndrome. The C allele of rs3212227/IL-12B confers risk to both BD and VKH. On the other hand, we failed to find a significant association between a set of seven other tested SNPs which belong to a group of three IL-12 related genes (IL-12B, IL-12Rβ1 and IL-12Rβ2) and either BD or VKH syndrome.

The data confirm previous associations between IL-12B gene polymorphisms and several autoimmune diseases such as ankylosing spondylitis and psoriasis [24], [25]. There is abundant evidence that immune response pathways in which IL-12 p40 (encoded by IL-12B) is involved may play an important role in the pathogenesis of uveitis. Previous reports have demonstrated that both Th1 and Th17 responses are augmented in Behcet's patients [35], [36]. The expression of IL-12p40 was shown to be higher in serum of BD patients [7]. Previous studies have also suggested that differences in the degree of IL-12p40 production may have an important impact on T cells reactivity [23]. As mentioned above, IL-12B polymorphisms have been reported to be associated with several immune disorders [24], [25]. In these studies an association was found for the SNP rs3212227 with AS and psoriasis.

Our data are not in agreement with preceding studies in BD from other ethnic populations. A small study with Turkish BD patients reported that the AC genotype and A allele frequency of rs3212227 were significantly higher in BD patients (BD cases: control = 80∶105, p = 0.015) [37]. Another small Japanese study did not find an association between rs3212227 and BD (BD cases: control = 55∶65) [38]. The difference between these studies and ours is that they were performed in BD patients with a different ethnic background and that the sample size was much lower.

Both the CC genotype and C allele of rs3212227/IL-12B were significantly increased in BD, whereas in VKH only the frequency of the C allele was significantly increased compared to the healthy controls. Statistical analysis did not show a significant association for the CC genotype as observed in the BD patients. Our findings add to the growing list of immune response gene mutations that are involved in the pathogenesis of both VKH and BD. Immune response genes such as HLA-DR4, IL-17 and STAT4 have been reported to be associated with VKH [15], [16], [17]. In BD gene mutations have been shown to be involved in disease susceptibility including human leukocyte antigen (HLA)-B51, IL-23R, DHCR7, and TLR-2 [11], [12], [13], [14].

Regarding the different genotypes of rs3212227 that confer susceptibility to various diseases in different ethnic groups, it has been reported that the A allele is a risk allele [39], [40]. On the other hand, there are other studies which describe the C allele as the risk allele [24], [41]. Differences between these results may due to the distinct nature of diseases in patients with a different ethnic background.

The effect of rs3212227 genotypes on the production of IL-12p40 by cultured cells is controversial. It has been reported that the rs3212227 CC/AC genotypes are associated with significantly decreased serum IL-12p40 levels [42]. However, there are other studies showing that different genotypes of rs3212227 do not mediate functional changes in IL-12p40 production [43], [44].

The exact mode of action of IL-12B rs3212227 gene polymorphisms and disease therefore remains unclear. Further functional studies are needed to clarify this issue.

We did not find significant associations with IL-12Rβ1 or IL-12Rβ2 and the two uveitis entities we studied. These two important receptors for IL-12, which have been reported to be associated with several autoimmune diseases [45], [46], play important roles in the immune response. It is possible that other SNPs in these three IL-12 related genes may play a role in uveitis. Further studies should be performed to address this issue.

There are a number of limitations in this study. We detected an association of rs3212227/IL-12B with BD and VKH patients in a Chinese Han population and our data cannot yet be extrapolated to other ethnic groups. BD and VKH syndrome are accompanied by various extraocular symptoms. Our patients were recruited by a department of ophthalmology and it is possible that our patients represent a subpopulation of these diseases. Analysis of patients from other medical departments should be carried out to rule out patient subgroup bias. Our data were confined to uveitis patients with BD and VKH and further studies should be carried out to investigate whether IL-12B is also involved in the pathogenesis of other uveitis entities. As mentioned above there might be differences in disease association in other ethnic patient populations and confirmation in other ethnic groups is necessary to solve this limitation.

In conclusion, this study shows an association between rs3212227/IL-12B polymorphisms with BD and VKH syndrome in a Chinese Han population, which supports a role for IL-12B in the development of intraocular inflammation in both BD and VKH syndrome.
